# Lu-177-PSMA-617 Prostate-Specific Membrane Antigen Inhibitor Therapy in Patients with Castration-Resistant Prostate Cancer: Stability, Bio-distribution and Dosimetry

**DOI:** 10.4274/mirt.08760

**Published:** 2017-06-01

**Authors:** Levent Kabasakal, Türkay Toklu, Nami Yeyin, Emre Demirci, Mohammad Abuqbeitah, Meltem Ocak, Aslan Aygün, Emre Karayel, Hüseyin Pehlivanoğlu, Nalan Alan Selçuk

**Affiliations:** 1 Istanbul University Cerrahpasa Faculty of Medicine, Department of Nuclear Medicine, İstanbul, Turkey; 2 Yeditepe University Faculty of Medicine, Department of Nuclear Medicine, İstanbul, Turkey; 3 Şişli Etfal Training and Research Hospital, Clinic of Nuclear Medicine, İstanbul, Turkey; 4 stanbul University Faculty of Pharmacy, Department of Pharmaceutical Technology, İstanbul, Turkey

**Keywords:** PSMA, prostate-specific membrane antigen, Lu-177-PSMA, prostate cancer, castration-resistant prostate cancer, radionuclide therapy

## Abstract

**Objective:**

The aim of the study was to estimate the radiation-absorbed doses and to study the *in vivo* and *in vitro* stability as well as pharmacokinetic characteristics of lutetium-177 (Lu-177) prostate-specific membrane antigen (PSMA)-617.

**Methods:**

For this purpose, 7 patients who underwent Lu-177-PSMA therapy were included into the study. The injected Lu-177-PSMA-617 activity ranged from 3.6 to 7.4 GBq with a mean of 5.2±1.8 GBq. The stability of radiotracer in saline was calculated up to 48 h. The stability was also calculated in blood and urine samples. Post-therapeutic dosimetry was performed based on whole body and single photon emission computed tomography/computed tomography (SPECT/CT) scans on dual-headed SPECT/CT system.

**Results:**

The radiochemical yield of Lu-177-PSMA-617 was >99%. It remained stable in saline up to 48 h. Analyses of the blood and urine samples showed a single radioactivity peak even at 24 hours after injection. Half-life of the distribution and elimination phases were calculated to be 0.16±0.09 and 10.8±2.5 hours, respectively. The mean excretion rate was 56.5±8.8% ranging from 41.5% to 65.4% at 24 h. Highest radiation estimated doses were calculated for parotid glands and kidneys (1.90±1.19 and 0.82±0.25 Gy/GBq respectively). Radiation dose given to the bone marrow was significantly lower than those of kidney and parotid glands (p<0.05) (0.030±0.008 Gy/GBq).

**Conclusion:**

Lu-177-PSMA-617 is a highly stable compound both in vitro and *in vivo*. Lu-177-PSMA-617 therapy seems to be a safe method for the treatment of castration-resistant prostate cancer patients. The fractionation regime that enables the longest duration of tumor control and/or survival will have to be developed in further studies.

## Abstract (tr)

**Amaç:**

Çalışmanın amaçları lutesyum-177 (Lu-177) prostat-spesifik membran antijen (PSMA)-617 tedavisinde absorbe edilen dozları hesaplamak, radyofarmasötiğin *in vivo* ve *in vitro* kararlılığını araştırmak ve farmakokinetik özelliklerini değerlendirmektir.

**Yöntem:**

Bu amaçla 7 hasta çalışmaya dahil edilmiştir. Hastalara 3,6 ila 7,4 GBq arasında değişen dozlarda ortalama 5,2±1,8 GBq Lu-177-PSMA-617 tedavi amacıyla verildi. Radyofarmasötiğin kararlılığı 48 saat süre ile serum fizyolojik içerisinde bekletilerek hesaplandı. Kararlılık ayrıca kan ve idrar örneklerinden de hesaplandı. Tedavi sonrası radyasyon dozu hesaplaması tüm vücut tek foton emisyon bilgisayarlı tomografi/bilgisayarlı tomografi görüntülerinden yapıldı.

**Bulgular:**

Lu-177-PSMA-617’nin radyokimyasal verimliliği >%99 olarak bulundu. Serum fizyolojik içerisinde 48 saat bozulmadan kaldı. Kan ve idrar örneklerinde yapılan yüksek performanslı sıvı kromotografisi ölçümlerinde 24 saat sonra bile tek bir zirve verdi. Dağılım ve eliminasyon fazlarının yarılanma zamanları sırasıyla 0,16±0,09 ve 10,8±2,5 saat olarak hesaplandı. Ortalama 24 saat ekskresyon hızı %56,5±8,8 olarak bulundu ve ekskresyon hızı %41,5’den %65,4’e kadar değişim gösterdi. Hesaplanan en yüksek radyasyon dozu parotis bezleri ve böbrekler için bulundu ve sırasıyla 1,90±1,19 and 0,82±0,25 Gy/GBq idi. Kemik iliğine verilen radyasyon dozu böbrekler ve parotis beziyle karşılaştırıldığında istatistiksel olarak çok düşüktü (p<0,05) (0,030±0,008 Gy/GBq).

**Sonuç:**

Lu-177-PSMA-617 *in vivo* ve in vitro şartlarda oldukça kararlı bir bileşik olarak gözükmektedir. Lu-177-PSMA-617 tedavisi kastrasyona dirençli prostat kanseri hastalarında kullanılabilecek oldukça güvenli bir radyofarmasötik olarak gözükmektedir. Hastalarda en uzun tümör kontrolü sağlayan fraksiyone tedavi yöntemi ileri çalışmalarla geliştirilmelidir.

## INTRODUCTION

Prostate-specific membrane antigen (PSMA) is a type 2 membrane glycoprotein that acts as a glutamate carboxypeptidase enzyme. It is highly expressed by all prostate cancers and its expression increases with increasing tumor aggressiveness (1,2,3). The unique expression of PSMA and ligand binding internalization of the PSMA via clathrin-coated pits and subsequent endocytosis makes it an excellent target for prostate cancer imaging and therapy using gallium-68 (Ga-68) and lutetium-177 (Lu-177) labeled ligands. Glu-NH-CO-NH-Lys-[Ga-68-(HBED-CC)] (Ga-68-PSMA-11) has been suggested as a novel tracer that can detect prostate cancer relapses and metastases with high contrast by targeting the PSMA (4,5,6,7,8). Also, therapeutic radiopharmaceutical Lu-177-PSMA-617 seems to be a promising novel tracer for systemic radionuclide therapy in patients with castration-resistant prostate cancer (9,10).

The basic principle of radionuclide therapy is to apply the maximum justifiable dose that does not cause serious toxicity in order to get an effective antitumor effect. The target organs for Lu-177-PSMA-617 therapy are the kidneys, parotid glands, and the bone marrow. In order to avoid toxicity, the amount of radiation dose given to target organs has to be estimated. Before introducing therapeutic applications we have performed a pre-therapy dosimetry study, and the initial results suggested that Lu-177-PSMA-617 therapy is a safe method (11). The target organs were the parotid glands rather than kidneys and bone marrow. The organ radiation doses were within acceptable ranges, however, there was a substantial individual variance that indicates that patient dosimetry is mandatory.

Therefore, we aimed to estimate the radiation-absorbed doses to dose limiting organs after systemic therapy with Lu-177-PSMA-617 in patients with castration-resistant prostate cancer. In addition, we also studied the *in vivo* and *in vitro* stability and pharmacokinetic characteristics of Lu-177-PSMA-617.

## MATERIALS AND METHODS

### Patients

In order to calculate the radiation absorbed doses, 7 patients were included into the study. All patients had histopathological diagnosis of prostate cancer. The ages ranged from 66 to 82 years (mean 71±5.2 years). Patients had prostatic surgery (n=2) and radiation therapy (n=5). All patients had androgen deprivation therapy and chemotherapy. All patients had increasing blood PSA levels, despite chemotherapy. The Gleason score was 9 in 4 patients, 8 in 3 patients. Blood PSA levels ranged from 4.2 to 219.0 ng/mL (mean 80.6±88.4 ng/mL). In order to decide the eligibility for Lu-177-PSMA-617 treatment all patients had Ga-68-PSMA-11 positron emission tomography/computed tomography imaging, and all patients had radiopharmaceutical uptake at the lesion site. All patients received a treatment of Lu-177-PSMA-617 with slow infusion in closed infusion equipment when the patients were in fasting state. The injected Lu-177-PSMA-617 activity ranged from 3.6 to 7.4 GBq with a mean of 5.2±1.8 GBq. The amount of Lu-177-PSMA-617 activity was decided empirically according to the tumor load of the patient in bones. Patients with widespread metastases in bones received a lower amount of radiopharmaceutical. Dosimetry calculations could be made in 6 patients due to missing data in one patient (Table 1). The study was approved by the Cerrahpaşa Medical Faculty Local Ethical Committee (protocol number: 830458809/604.01/02-268589).

### Preparation of Lu-177-PSMA-617

The radiolabeling of PSMA-617 (10) was performed in a hotcell using Lu-177 Cl_3_ (47 MBq/nmol of ligand) in 0.05 mol L^-1^ HCl (Perkin Elmer, USA) with sodium ascorbate buffer pH 4.5 (Polatom, Otwock-Swierk Poland) at 95 °C for 15 minutes. After cooling down of the reaction vial to room temperature the volume was adjusted to 2 mL with saline and 0.5-1.0 mL of sterile DTPA solution (3 mg mL^-1^ DTPA in saline) was added. After sterile filtration of this preparation to a sterile vial the volume was completed to 20 mL with sterile saline under aseptic conditions. Radiochemical purity was determined by instant thin layer chromatography (ITLC)-silica gel and radio-high performance liquid chromatography (HPLC) and was found as ≥98%.

### Stability of Lu-177-PSMA-617

The prepared patient dose of Lu-177-PSMA-617 (3.7 GBq) was incubated in saline at 37 °C up to 48 h. At determined time points incubation solution sample was injected to the reversed-phase (RP)-HPLC for evaluating the *in vitro* stability of the patient dose up to 48 h. In 7 patients *in vivo* stability was checked by using blood samples obtained at 0-3, 30, 60, 120, 180 min and using urine samples obtained up to 24 h after injection of Lu-177-PSMA-617. Blood samples received from patients were precipitated with acetonitrile (1:1) and then vortexed. The precipitate was separated by centrifugation (5 min at 14680 rpm). For the RP-HPLC analysis, the supernatant was diluted with bi-distilled water (1:1), filtered and then injected into RP-HPLC. Collected urine samples from patients were diluted with bi-distilled water, filtered and immediately analyzed by RP-HPLC. Excreted urine of each patient was collected for 24 hours and 10 mL urine samples were measured in a dose calibrator and excretion rate of radiopharmaceutical was calculated.

### Imaging and Dosimetry

Post-therapeutic dosimetry was performed based on whole body and single photon emission computed tomography/computed tomography (SPECT/CT) scans on dual-headed Symbia T16 SPECT/CT system (Siemens Medical Solutions, Erlangen, Germany) with 3/8-inch crystal thickness. Whole body scans (WBS) were performed using medium energy parallel hole collimators at time marks of 4, 24, 48 and 120 hours after administration of the prescribed treatment activity. Scan parameters include single energy peak at 208 keV with window width of 15%, 256×1024 matrix size with pixel size of 2.4×2.4 mm2, 25 cm/min scan speed. Triple energy window-scatter correction (TEW-SC) was applied to all images using lower and upper scatter energy windows at 180 keV and 235 keV with window widths of 10%.

Two-bed SPECT/CT scan for each patient was performed only after 24^th^ hour WBS to avoid unnecessary exposure to CT due to subsequent scans. Matrix size in SPECT imaging was 128×128 with pixel size of 4.8×4.8 mm^2^. Counts were collected in a non-circular orbit during 25 seconds per view and total of 96 views (48 for each head) per bed position. To correct photon attenuation, CT scan with 5 mm slice thickness were acquired after SPECT scan using parameters of 130 kVp and 60 mAs per slice. SPECT images were reconstructed using iterative reconstruction algorithm with collimator-detector response compensation (OSEM Flash 3D, 4 iterations, 8 subsets). TEW-SC was also applied with the parameters stated above. Count-to-activity calibration of SPECT images was performed using NEMA IEC Body Phantom. The larger fillable sphere in the phantom was filled with the solution of Lu-177 with activity concentration of 185 kBq/mL. The same acquisition and reconstruction parameters that has been used for patient examinations were employed. A volume of interest (VOI) was drawn around the sphere in 3D with a threshold of 40% of maximum count. To obtain the calibration factor, total counts in the VOI were equalized to the total activity in the sphere.

Kidneys, liver, parotid gland and the rest of the body were selected as source organs. Region of interests were drawn over the source regions on anterior and posterior WBS images for all time points. Total counts for each source organ were determined by conjugate view method with geometric background subtraction as described in MIRD Pamphlet No.16 (12). To perform geometric background subtraction, required body and mean organ thicknesses were determined from CT images of the SPECT/CT scan. Total activity in source organs at 24 hours after p.i. were determined from SPECT images by drawing VOIs around the source organ in 3D with the threshold of 40% of central mean counts of each organ. Counts in the VOIs were converted to activity by multiplication with the calibration factor described above. A conversion factor for each source organ was obtained from quotient of the organ counts of the 24^th^ hour WBS to the activity in the organ determined from 24^th^ hour SPECT imaging to convert WBS counts to activity for the other time points.

OLINDA/EXM (version 1.1) software was used to calculate the absorbed dose according to MIRD scheme. Both male and female adult phantoms were used. The source organ masses determined from CT slices as well as the total body mass were adjusted in the software. As the parotid glands are not included in the phantoms, unit density sphere model was used to estimate self-dose to the glands. Time integrated activity coefficients were determined using fit functions of the software.

Absorbed dose to the bone marrow was calculated from blood samples at different time points of 3, 10, 20, 40, 60 and 90 minutes, 2, 3, 24, 48 and 72 hours after the start of Lu-177-DKFZ-617 infusion, and the whole body activity was determined from the WBSs according to the EANM Guideline (13). The red marrow to blood ratio of 1 was selected for calculations.

### Statistical Analysis

All results were expressed as mean±SD. For statistical analysis, a dedicated statistical software was used (StatPlus:mac v5. AnalystSoft Inc. BC. CA). Wilcoxon test was used to compare different groups. A p value lower than 0.05 was considered as significant.

## RESULTS

### Stability and Bio-distribution

**Figure 1:**
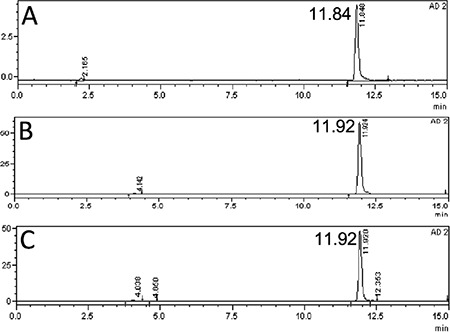
Reversed-phase-high performance liquid chromatography (RP-HPLC) profiles of Lu-177-PSMA-617 (the RP-HPLC elution time of radioligand is in between 11.7-11.96 min) in 100 mCi patient dose incubated in saline at 37 °C A) at 0-3 min, B) at 24 h, C) at 48 h

**Figure 2:**
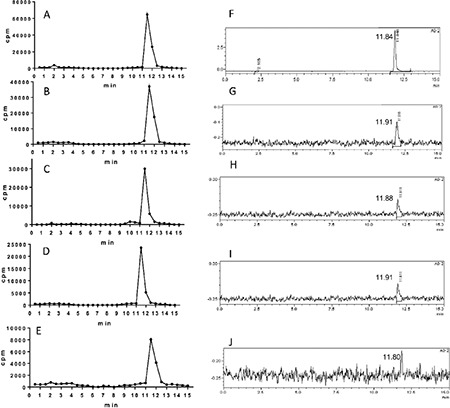
Reversed-phase-high performance liquid chromatography (RP-HPLC) profiles of Lu-177-PSMA-617 (the RP-HPLC elution time of radioligand is in between 11.7-11.96 min) in blood after 100 mCi injection of radioligand in a patient [A) 0-3 min, B) 30 min, C) 60 min, D) 120 min, E) 180 min] and 200 mCi injection of radioligand [F) 0-3 min, G) 30 min, H) 60 min, I) 120 min, J) 180 min]

**Figure 3:**
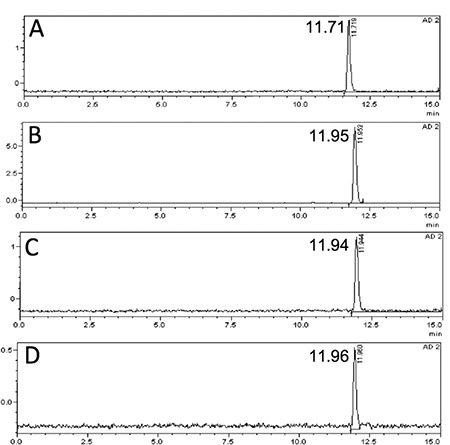
Reversed-phase-high performance liquid chromatography (RP-HPLC) profiles of Lu-177-PSMA-617 (the RP-HPLC elution time of radioligand is in between 11.7-11.96 min) in urine after 200 mCi injection of radioligand in a patient A) at 3 h, B) at 5 h, C) at 15 h, D) at 24 h

**Figure 4:**
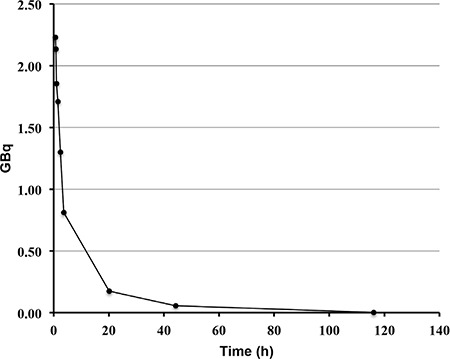
Mean blood-time radioactivity curve of all patients

The radiochemical yield of Lu-177-PSMA-617 was >99% by RP-HPLC and 98.7±0.97% by ITLC. The radiopharmaceutical remained stable in saline up to 48 h after preparation, and radiochemical purity was found >98% at 48 h incubation time point (Figure 1). RP-HPLC analyses of the blood and urine samples showed a single radioactivity peak corresponding to Lu-177-PSMA-617 even at 24 hours after injection (Figure 2 and 3). There was no other peak corresponding to metabolized Lu-177 PSMA-617. Blood time activity curve showed a rapid bi-exponential clearance curve (Figure 4). Half-life of the distribution phase was calculated to be 0.16±0.09 hours and the half-life of elimination phase was calculated to be 10.8±2.5 hours. Total body residence time showed great variation among patients and it was ranged from 23.1 hours to 44.0 hours with a mean value of 37.5±7.5 hours. Almost half of the injected amount of radiopharmaceutical was excreted within 24 hours. The mean excretion rate of injected radiopharmaceutical amount was 56.5±8.8%, ranging from 41.5% to 65.4%.

### Toxicity

Patients were followed for 24 hours after infusion of radiopharmaceutical within 15 minutes. All patients tolerated the procedure very well and we did not observe any acute side effect. We did not observe any change in blood pressure, heart rate or body temperature. We did not observe any change in complete blood counts within one week.

### Dosimetry

**Figure 5:**
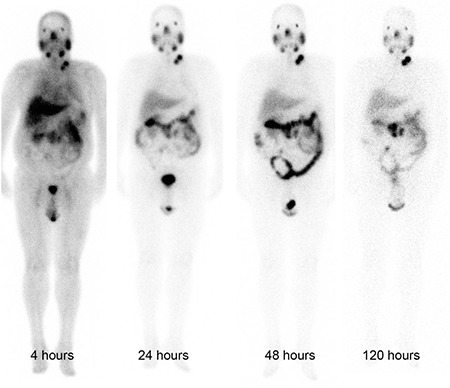
Lutetium-177 prostate-specific membrane antigen-617 whole body anterior images of a patient at different time points

In whole body images obtained 4 hours p.i. there was high blood pool along with soft tissue uptake (Figure 5). Because of a rapid clearance from the blood, intense radiopharmaceutical uptake at the physiological uptake sites and at the sites of tumor lesions was observed in images obtained later. The radiopharmaceutical uptake decreased considerably in images obtained at 120 hours p.i.

The calculated radiation absorbed doses for each organ showed great variance among patients. The highest radiation estimated doses were calculated for the parotid glands and kidneys. For parotid glands the calculated mean radiation absorbed dose per GBq was 1.90±1.19 Gy. For the kidneys, the mean radiation absorbed dose was calculated to be 0.82±0.25 Gy/GBq. For the bone marrow, calculated radiation dose was significantly lower than those of kidney and parotid glands (p<0.05). The calculated radiation dose to the bone marrow was 0.030±0.008 Gy/GBq (Table 1).

The estimated maximum safe activities, at which organ doses do not exceed radiation absorbed dose constraints for parotid glands, kidneys and bone marrow, were calculated to be 21.7±12.8 GBq, 32.9±19.2 GBq and 73.8±27.1 GBq, respectively (Table 2).

## DISCUSSION

The radiolabelling procedure of Lu-177-PSMA-617 is easy and it gives consistent high radiolabelling yields. Its radiochemical purity was over 98%. It remains stable in saline up to 48 h after radiolabelling, which provides time for quality control, transport of radiopharmaceutical from laboratory to the ward, and allows making a flexible therapy plan within the ward. It is also possible to make a slow infusion, if needed, for the administration of radiopharmaceutical to the patient. Administration to the patient was safe without any acute side effects and the patients tolerated it very well. After the administration, it is rapidly distributed within the body giving a bi-exponential blood clearance curve, which was consistent with the finding obtained in the pre-therapeutic dosimetry study published previously (11). Radiopharmaceutical also remained stable in blood and was excreted without any degradation. More than half of the administered radioactivity is excreted through the kidneys. During the first 24 hours, good hydration and frequent urination may protect the bladder from high radiation-absorbed dose. Due to its rapid excretion, it seems that Lu-177-PSMA-617 therapy can be given in an outpatient protocol according to national regulations, since 6 h after administration of the radiopharmaceutical the dose rate decreases to 20 μSv/h and the radiation exposure of caregivers remains below 5 mSv (14).

For the kidney, parotid gland and bone marrow, calculated radiation-absorbed doses were 0.82, 1.90 and 0.03 Gy/GBq respectively. These results are comparable with the pre-therapeutic dosimetry findings (11). The minor differences may be because of the different methodologies used between two studies, because 3D methods are not affected from the overlapping structures. Our results seem to be higher than the results of Delker et al. (15). They calculated the radiation-absorbed doses for kidney, salivary glands, and bone marrow as 0.6, 1.41 and 0.012 Gy/GBq, respectively. This difference can be attributed to the different time periods used for the calculation of absorbed doses. Delker et al. (15) have used a shorter time period for obtaining data and estimating residence time, which was only 72 h after injection as compared to our data, which was containing the data of 110 h after administration of radiopharmaceutical.

In accordance with the previous bio-distribution and dosimetry studies, the highest radiation absorbed dose was observed in salivary glands due to high uptake of the radiopharmaceutical and it seems to be the critical organ at risk rather than the kidney and bone marrow (11,12,13,14,15). Xerostomia is a frequent side effect of radiation therapy after exceeding 40 Gy of radiation doses, which decreases patient’s quality of life (16,17). On the other hand, although salivary gland dysfunction is a common finding in patients treated with radioiodine, it is usually transient and persistent dysfunction rate is reported to be only 5% (18). As stated by Delker et al. (15), we also did not observe xerostomia in patients treated with Lu-177-PSMA-617 during follow-up.

Bone marrow absorbed dose is the major factor that limits radiopharmaceutical dose given to the patient and may have fatal consequences. The radiation absorbed dose given to the bone marrow is 0.030±0.008 Gy/GBq. In order not to exceed the 2 Gy limit to develop bone marrow toxicity, it seems that it is safe to administer up to 73.8 GBq (19). We agree with Delker et al. (15) that bone marrow toxicity seems to be unlikely with the suggested amounts of Lu-177-PSMA-617 for each cycle. However, bone marrow dose estimation in patients with prostate cancer which have extensive bone metastases using blood based dosimetry models may underestimate the absolute dose due to the existence of high radiopharmaceutical avid lesions which increase the dose delivered to bone marrow. Moreover, end-stage prostate cancer patients are extensively treated with chemotherapy and radiotherapy, which may potentially increase the risk of development of hematotoxicity even with a lower amount of radiation dose to the bone marrow.

The kidneys are important target organs due to their high radiotracer uptake and excretion. Based on the earlier experience obtained from conventional external beam radiotherapy, the maximum kidney dose is generally accepted as 23 Gy (19). In order to reach this dose limit to the kidneys a mean of 32.9 GBq of Lu-177-PSMA-617 can be given. It seems that it is safe to administer usual doses without developing kidney toxicity.

## CONCLUSION

In conclusion, radiolabelling of Lu-177-PSMA-617 is easy, and it is a highly stable compound both *in vitro* and *in vivo*. Lu-177-PSMA-617 therapy seems to be a safe method for the therapy of castration-resistant prostate cancer patients. The fractionation regime that enables the longest duration of tumor control and/or survival will have to be developed in further studies. It shows substantial difference among patients. Therefore, a patient specific dosimetric approach should be applied before therapy to prevent organ toxicity.

## Figures and Tables

**Table 1:**
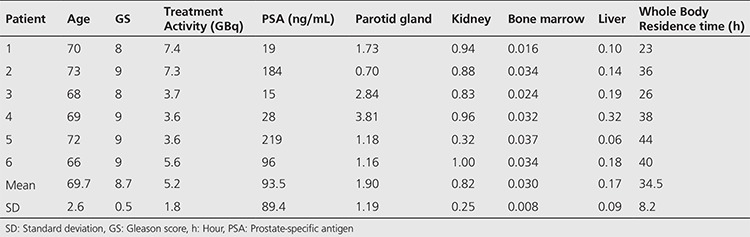
Patient characteristics and calculated radiation absorbed doses (Gy/GBq of Lu-177-PSMA-617) per organs

**Table 2:**
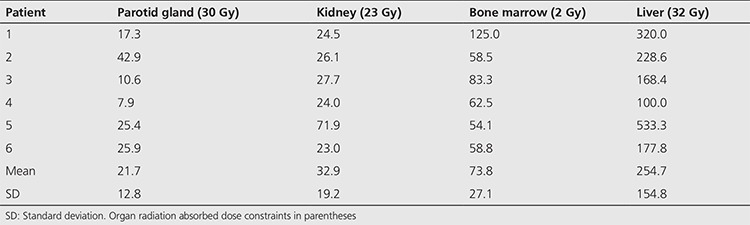
The calculated amount of radiopharmaceutical (GBq) to exceed radiation absorbed dose limits of organs at risk
